# Needs of Adolescents and Young Adults with Neurodevelopmental Disorders: Comparisons of Young People and Parent Perspectives

**DOI:** 10.1007/s10803-017-3295-x

**Published:** 2017-09-11

**Authors:** Hanna Eklund, James Findon, Tim Cadman, Hannah Hayward, Declan Murphy, Philip Asherson, Karen Glaser, Kiriakos Xenitidis

**Affiliations:** 1 Department of Forensic and Neurodevelopmental Science, Institute of Psychiatry, Psychology & Neuroscience, King’s College London, De Crespigny Park, London, SE5 8AF UK; 2 MRC Social Genetic and Developmental Psychiatry, Institute of Psychiatry, Psychology & Neuroscience, King’s College London, De Crespigny Park, London, SE5 8AF UK; 3 Department of Social Sciences, Health and Medicine, Institute of Gerontology, King’s College London, Strand, London, WC2R 2LS UK

**Keywords:** Neurodevelopmental disorders, ADHD, ASD, Needs assessment, Young adulthood

## Abstract

This study used the Camberwell Assessment of Need for adults with Developmental and Intellectual Disabilities (CANDID) to examine the social, physical health and mental health needs of 168 young people (aged 14–24 years) with neurodevelopmental disorders and compared young person and parent ratings of need. Agreement was poor in 21 out of 25 domains. Parents consistently reported higher levels of need than young people in the majority of domains although young people with ADHD reported significantly more needs in *physical health, eyesight/hearing, seizures, other mental health problems* and *safety of others* than their parents. Both parent and young person perspectives of needs are necessary to ensure that needs that are predictive of current or future poor outcomes are not missed.

## Introduction

Government strategies place patients at the heart of the NHS and emphasise the importance of tailoring services to meet the needs and preferences of patients, their families and their carers (Jivanjee et al. 2008; Jivanjee and Kruzich 2011; Department of Health 2013). An example of this drive towards more needs-led health services can be seen throughout the White Paper Equity and Excellence: Liberating the NHS which promised an NHS that puts patients at its centre, offers patients greater choice and control, and provides personalised care that reflects individual needs (Department of Health 2010b). The paper recognised that in order to meet the needs of children, young people, families and carers, their own perceptions are important for prioritising services (Department of Health 2010a). Moreover, the more recent Government ‘Young People’s Mental Health Taskforce’ report stated that mental health support should be provided based on the young person’s presenting needs and the level of professional or family concern, not just clinical diagnosis (Department of Health 2015).

However, for many young people with neurodevelopmental disorders health service provision continues to be poor, particularly at the transition to adulthood (Marcer et al. 2008; Eklund et al. 2016; Taylor et al. 2010; NICE 2012, 2008; Singh and Tuomainen 2015). There is increasing recognition that both ADHD and ASD can persist into adulthood and are associated with significant costs and burdens (McGovern and Sigman 2005; Faraone et al. 2006; Biederman et al. 2012; NICE 2008, 2012). However, young adults with ADHD and ASD across the country are often deemed not to meet criteria for adult mental health services on the basis of ‘severe and enduring mental illness’. Transition arrangements between child and adult health services are often of poor quality (Singh 2009), and are experienced negatively by young people and parents (Butterworth et al. 2016; Fegran et al. 2014; Eklund et al. 2016). Subsequently many young people with neurodevelopmental disorders experience discontinuity of care and exacerbation of distress or co-existing mental illness (Singh 2009; Butterworth et al. 2016) highlighting the significant challenges parents are likely to encounter when trying to support their children during the transition to young adulthood (Cadman et al. 2012).

Within this context, a *needs-led* approach to mental health-care planning and outcome evaluation seems particularly useful, since it allows the consideration of multiple perspectives of those involved in the care process (McCrone and Strathdee 1994). When needs are assessed from multiple perspectives this is likely to lead to a better relationship between clinician and service-user (Lasalvia et al. 2012). Moreover, it is likely to help identify self-perceived health and social needs and discrepancies that may inform clinical practice and lead to a more coherent care plan (The National Audit Office 2009; Islam et al. 2016).

Currently information regarding perceptions of need (as opposed to symptoms and impairments) among young people with ADHD and ASD is lacking. Studies of need in generic mental health populations have shown that ratings of needs differ significantly between assessors; carers tend to rate more needs than patients, particularly in the areas of home and self-care (Hancock et al. 2003; Lasalvia et al. 2012). Those studies that have assessed needs in people with severe mental illness which have considered the perspectives of patients (service users) and staff have shown that patients and staff rate a broadly similar number of needs but in different areas (Appleton and Pugh 2011; Coleman and Berenson 2004; American Academy of Child and Adolescent Psychiatry 2007). Patient and staff perspectives have also been compared to carer perspectives in people with mental health problems which have reported that carers report higher levels of need, particularly in the care of home and self-care, than either patients or staff (Hancock et al. 2003; Macpherson et al. 2008).

To our knowledge, no studies to date have provided a detailed investigation of needs among adolescents and young adults with neurodevelopmental disorders or the relationship between needs assessed by parents and young people. Only one study reported on the needs of adult substance use disorder (SUD) patients with and without co-occurring attention deficit hyperactivity disorder (ADHD) or autism spectrum disorder (ASD). This study, which used the Camberwell Assessment of Needs (CAN) to identify care needs, reported that SUD patients have fewer care needs than SUD patients with co-occurring ADHD or ASD and that the SUD and SUD + ADHD groups report needs in similar domains. The study also found that the SUD + ASD group showed a greater number of and more extensive care needs (Kronenberg et al. 2015).

The present study is the first to comprehensively assess the health and social needs of adolescents and young adults with neurodevelopmental disorders according to the perspectives of both the young person and their parent, thus addressing a gap in this domain. We hypothesised that, like other young people with a diagnosed mental health disorder, adolescents and young adults with neurodevelopmental disorders experience a complex mixture of health and social needs and are likely to experience problems that are not directly disorder-related such as relationship problems and daily difficulties.

## Methods

Data were collected as part of a prospective study examining needs and service use among young people with a childhood diagnosis of ADHD or ASD at transition from adolescence to young adulthood (aged 14–24). Face-to-face interviews with parent and young person were carried out using the Camberwell Assessment of Need for adults with Developmental Disabilities (CANDID), a standardised instrument for the assessment of needs of adults with learning disability and mental health problems (Xenitidis et al. 2000). Our aim was to compare the perceptions of need between young people and their parents.

### Sample

We included 168 families consisting of young people aged 14 to 24 years (n = 85 with ASD and n = 83 with ADHD) and their parents (mostly mothers; 95%; n = 85 with ASD and n = 83 with ADHD). Families were recruited through their child’s childhood clinical diagnosis of an ASD or ADHD from Child and Adolescent Mental Health Services (CAMHS) and adult clinics, charities, and research databases that form part of our clinical research networks. Clinical diagnosis of autism was confirmed in all cases using the Autism Diagnostic Interview—Revised and ADHD (combined type) was defined using the *DSM-IV* criteria. As participants in the ADHD group were originally recruited for the International Multi-Centre ADHD Genetics (IMAGE) project, they were excluded if they had been diagnosed with autism, epilepsy, general learning difficulties, brain disorders, or any genetic or medical disorder associated with externalizing behaviours that might mimic ADHD based on both history and clinical assessment.

### Assessment Tool

There is currently no “gold-standard” measure for assessing needs in young people with neurodevelopmental disorders at transition from childhood to adulthood. However, given that ADHD and ASD are both developmental disorders, are often associated with intellectual (learning) disabilities and mental health problems The Camberwell Assessment of Need for Adults with Developmental and Intellectual Disabilities (CANDID) was judged as the most appropriate of the available needs assessment instruments. The CANDID (Xenitidis et al. 2000) was developed by modification of the Camberwell Assessment of Need (CAN) (Phelan et al. 1995) the most widely used needs assessment tool in general mental health services. The CANDID assesses the presence of met and unmet needs in 25 domains, as shown in Table 2.

For each domain, examples are given of what constitutes a need, and a presence of need is rated on a three-point scale: 0 = no need, 1 = met need (“no or moderate problem because of continuing intervention”), and 2 = unmet need (“current serious problem”). A rating of 9 is used for “not known”. The needs of the patient can be assessed separately by the patient a member of staff, and an informal carer (e.g. a parent). The development and psychometric properties of the instrument have been examined and reported previously (Xenitidis et al. 2000).

### Statistical Analysis

Data analysis was carried out with the Statistical Package for the Social Sciences (SPSS) Windows Version 21. The total number of needs, met needs and unmet needs identified by parents and young people were calculated from the need ratings for each domain. As the data were not normally distributed, comparisons between parent and young person ratings were initially performed using the Mann–Whitney *U* test but given that the results of parametric tests were the same only the parametric results (*t*-tests) are reported here. Pearson’s correlation tests were used to analyse bivariate associations between rater dyads. Using the standard approach to dealing with CAN data (Slade et al. 1996), absence of need was defined as a CANDID rating of 0 or 9, and presence of need was defined as a rating of either 1 or 2. In accordance with Landis and Koch (1977), we linked Kappa coefficients to level of agreement as follows: 0–0.2 “poor” agreement; 0.2–0.4 “fair” agreement; 0.4–0.6 “moderate” agreement; 0.6–0.8 “substantial” agreement; 0.8–1.0 “almost perfect” agreement (Simon et al. 2009).

## Results

### Sample Characteristics

A total of 168 participants provided complete paired (i.e. young person and parent) data on needs (ADHD = 83, ASD = 85). Young people had an average age of 17.6 (range 14–24, s.d. = 2.5) and consisted mostly of males (91%) who were in full-time or part-time education (72%) and were still living at home (88%). 15% of the ASD group had a general learning disability compared to none in the ADHD group. Parents were mostly mothers (96%) who reported being in paid employment (67%). At the time of assessment 57% of young people reported being in contact with services for their condition. We compared the two subgroups on social-demographic variables and found only two significant difference with respect to parental education (64% of ADHD parents were highly educated versus 42% of ASD parents: χ^2^ (1, *N* = 163) = 7.720, p < 0.005) and young person medication use (34% of ASD on medication versus 49% of young people with ADHD χ^2^ (1, *N* = 162) = 3.865, p < 0.05).

### Total Needs

Table 1 shows the mean total number of met and unmet needs as assessed by young person and parent. There were significant differences in ratings of *total number of needs* between young person and parent with the parents reporting on average 2.92 more needs than their child (95% CI 2.44–3.40, p < 0.001). Parents reported significantly more *met needs* (95% CI 1.28–1.99, p < 0.001) and *unmet needs* (95% CI 1.28–1.99, p < 0.001) than young people.

**Table 1:** Mean total number of needs, met needs and unmet needs, and difference between assessors

	Assessment of need Mean (SD) (95% CI)		Difference in assessors Mean (95% CI)
Assessor	Young person	Parent	Young person–parent
n	168	168	
Total needs	4.29 (3.05) (3.83–4.75)	7.21 (4.19) (6.58–7.85)	2.92 (df = 167, t = 11.922*) (2.44–3.41)
Met needs	2.72 (2.42) (2.36–3.09)	4.35 (3.09) (3.88–4.82)	1.63 (df = 167, t = 9.060*) (1.28–1.99)
Unmet needs	1.57 (1.88) (1.29–1.86)	2.86 (2.76) (2.45–3.28)	1.29 (df = 167, t = 6.902*) (0.92–1.66)

A significant interaction effect between assessor and diagnostic group was found (*F* = 14.567, df = 1, 166; p < 0.001) however this result were not significant when *total need* was added as a covariate

*T*-test, 2 tailed, *p < 0.0001

The difference between assessors was compared by diagnostic subgroup using a mixed model analysis of variance (ANOVA). For *total number of needs*, a main effect of diagnostic group was observed (*F* = 65.531, df = 1, 166; p < 0.001) with a mean of 7.48 needs reported in the ASD group and 3.98 in the ADHD group. There was a significant interaction effect between assessor and diagnostic group (*F* = 14.567, df = 1, 166; p < 0.001) suggesting a greater difference between assessors in the ASD group, however these results were not significant when total need was added as a covariate. A main effect of diagnostic group was also observed for *total met needs* (*F* = 90.979, df = 1, 166; p < 0.001) with a mean of 5.01 reported total met needs in the ASD group compared to 2.02 total met needs in the ADHD group, however these results were not significant when total need was added as a covariate. A significant interaction effect between assessor and diagnostic group (*F* = 11.320, df = 1, 166; p < 0.001) suggested a greater difference between assessors within the ASD group. No main effect of diagnostic group (*F* = 2.728, df = 1, 166; p = 0.1) or interaction (with and without adding total need as a covariate) between assessor and diagnostic group (*F* = 2.810, df = 1, 166; p = 0.096) was observed on *total unmet needs*.

### Individual Needs

On examining individual need domains the number of young people and parent rating a need as present (either met or unmet) in each domain is shown in Table 2, together with the level of agreement for each dyad. The agreement indicated by a kappa coefficient can be slight (up to 0.2), fair (0.21–0.4), moderate (0.41–0.6), substantial (0.61–0.8) or almost perfect (0.81–1.0) (Landis and Koch 1977). The mean Kappa coefficient for presence of need was fair (0.25) however there was only slight agreement in ratings of met needs (0.13) and unmet needs (0.084). There were some striking differences in the amount of needs identified within particular domains, with parents rating significantly more needs than young people across all domains. Notably, 55% of parents rated a need in the *exploitation risk* domain in the absence of a young person rated need. In the *food* domain 40% of parents rated a need in the absence of a young person rated need, and in the *money budgeting* domain, 34% of parents rated a need in the absence of young person rated need. Within the ASD subgroup, parents rated more needs across all domains than young people. Within the ADHD subgroup, the young people rated more needs than parents in the following domains: *physical health, eyesight/hearing, seizures, other mental health problems* and *safety of others*.

**Table 2:** Young person (YP) and parent (P) ratings of presence of need in individual domains, and level of agreement between assessors

Domain	Presence of need identified		Agreement on presence of need 95% CI	Agreement on presence of met need 95% CI	Agreement on presence of unmet need 95% CI
	YP n (%)	P n (%)	Kappa (%)	Kappa (%)	Kappa (%)
Accommodation	0 (0)	5 (3.0)	* (97)	* (98.2)	*(98.8)
Food	14 (8.3)	81 (48.2)	0.129 (57.7)	0.65 (61.9)	−0.059 (87.5)
Looking after home	39 (23.4)	90 (53.9)	0.115 (53.9)	0.047 (53.3)	−0.066 (87.4)
Self-care	28 (16.8)	75 (44.9)	0.294 (67.1)	0.087 (60.5)	−0.046 (88.6)
Daytime activities	24 (14.3)	56 (33.3)	0.281 (72.6)	−0.005 (83.9)	0.304 (81.5)
Physical health	33 (19.6)	39 (23.2)	0.365 (78.6)	0.194 (80.4)	0.184 (86.7)
Eyesight/hearing	56 (33.3)	61 (36.3)	0.672 (85.1)	0.403 (76.2)	−0.043 (82.7)
Mobility	3 (1.8)	5 (3)	0.744 (98.8)	0.323 (97.6)	* (99.8)
Seizures	4 (2.4)	6 (3.6)	0.588 (97.6)	−0.016 (96.4)	−0.012 (97.6)
Major MHP	5 (3)	15 (9)	0.372 (92.8)	* (97.6)	0.478 (95.2)
Other MHP	47 (28)	72 (42.9)	0.250 (64.9)	0.133 (75)	0.028 (70.8)
Information	25 (15.1)	54 (32.5)	0.187 (69.3)	0.067 (81.9)	0.110 (78.9)
Exploitation risk	8 (4.8)	99 (59.6)	0.025 (42.8)	0.058 (61.4)	−0.034 (78.9)
Safety to self	5 (3.)	26 (15.6)	0.083 (83.8)	* (91.0)	0.087 (91.6)
Safety to others	30 (17.9)	42 (25)	0.263 (75)	0.227 (88.1)	0.112 (79.8)
Inappropriate behaviour	25 (14.9)	70 (41.7)	0.124 (61.3)	−0.033 (79.8)	0.120 (73.2)
Substance misuse	3 (1.8)	9 (5.4)	0.315 (95.2)	0.394 (98.2)	0.273 (97)
Communication	23 (13.9)	62 (37.3)	0.130 (64.5)	0.012 (72.3)	0.032 (82.5)
Social relationships	24 (14.3)	73 (43.5)	0.278 (67.3)	0.023 (81.5)	0.089 (70.2)
Sexual relationships	3 (1.8)	7 (4.2)	0.179 (95.2)	−0.012 (97.6)	−0.010 (96.4)
Caring for someone	2 (1.2)	3 (1.8)	−0.014 (97)	−0.008 (98.2)	−0.006 (98.8)
Basic education	33 (19.8)	56 (33.5)	0.327 (73.1)	0.257 (83.8)	0.230 (80.8)
Transport	19 (11.3)	55 (32.7)	0.383 (77.4)	0.007 (72)	−0.020 (87.5)
Money budgeting	43 (25.7)	100 (59.9)	0.137 (52.7)	−0.008 (62.3)	0.232 (74.9)
Welfare benefits	14 (8.5)	44 (26.8)	0.010 (69.5)	0.071 (90.9)	−0.024 (76.2)
Mean kappa			0.259	0.131	0.085

*Kappa coefficient could not be calculated due to insufficiently spread data

## Discussion

This study evaluated, for the first time, self-perceived needs among adolescents and young adults with neurodevelopmental disorders and as perceived by their parents and the level of agreement between young person and parent ratings. This is significant because in order to meet the needs of young people with neurodevelopmental disorders, their own perceptions of need are important for prioritising services and should be taken into account, especially if these differ significantly from that of their parents (Department of Health 2010a; Fegran et al. 2014). We found that young people were less likely than their parents to rate a need as present in most domains. Young people rated lower overall levels of needs than parents; agreement between young people and parents varied across CANDID domains, being generally lower in the domains that did not involve physical health needs.

Nevertheless, this study underscores the importance of the views of young people themselves, as well as assessments by parents. For example, we found that young people with ADHD were more likely to report needs in *physical health, eyesight*/*hearing, seizures, other mental health problems* and *safety of others* than their parents. While such disagreements between young people and parents may not be surprising considering the influence of age, typical adolescent development and findings from previous studies in general mental health populations that have found significant differences between service user and carer rated needs (Hancock et al. 2003; Macpherson et al. 2008), the results of our study provide empirical and specific data on where there is and isn’t agreement on young people and carer rated needs among people with ASD and ADHD. It is important that the views of young people but also that of their parents are always recorded and that any information regarding unmet needs are followed-up in clinical practice. For instance, at an individual level, once needs are identified as unmet this information can be used in individual care planning (e.g. making a referral to a speech and language therapist of a young person with an unmet need identified in the communication domain). At a population (local, regional, national) level, aggregated CANDID data can inform allocation of resources (e.g. employment of a clinical psychologist in an area with high unmet need identified in the relevant domain).

### Differences in Ratings of Total Number of Needs

Young people assessed their total number of needs as lower than parent assessments. On the one hand, this finding is consistent with cognitive deficits and lack of insight often reported among young people with neurodevelopmental disorders (Barkley et al. 2011; Henry et al. 1994) and the evidence that young people with neurodevelopmental disorders frequently under-report their symptoms (Barkley et al. 2002). However, it is also important to bear in mind that differences between assessors are common (Hancock et al. 2003; Macpherson et al. 2008) and are in fact some of the most consistent effects observed in clinical science (Achenbach et al. 1987). Hence, differences in ratings of need found in our study may reflect differences among typically developing youth and their parents rather than differences among youth with neurodevelopmental disorders and their parents per se.

The pair-wise comparison of met and unmet needs indicated that the difference between the young person and parent rating was due to differences in both met and unmet need ratings, with parents rating significantly higher levels of both met and unmet needs. This may reflect a more limited awareness among young people of the type and amount of help they receive, and parents being more aware of the services and treatments available. Given that most young people in this study were still living at home, it may be reasonable to expect parents to have good awareness of needs that are reflected in impairments within the home environment and other environments in which the parent may be able to observe their child regularly. Carers may also be more likely to rate need, especially met need, in the areas that they have had (or continue to have) more involvement in managing. In addition, parents may be more likely to rate their own interventions positively. However, given that high levels of carer burden have been reported in this group and that higher levels of carer burden appear to be predicted by parental appraisal of their children’s unmet needs (Faraone et al. 2006) it may be that parents who are very burdened are actually less able to accurately appraise their children’s needs.

### Differences in Ratings of Individual Needs

There were some interesting differences between young people and parents in ratings of individual domains. Parents tended to perceive problems with everyday functioning such as looking after the home and self-care which young people did not. Parents also reported higher levels of need in exploitation risk and inappropriate behaviour, possibly due to increased awareness of such needs or increased concern for their child’s welfare. Conversely, we found that young people with ADHD were more likely than their parents to perceive a risk to others and to report a problem with common mental health problems, such as depression and anxiety. Such disagreements in ratings of individual needs may not be surprising given that young people and parents are likely to attach different levels of importance to different life domains. It may be that the young persons are particularly concerned about the impact of hurting someone else or an additional mental health problem on their future success as an independent adult whereas parents may be more concerned about the risk of exploitation and their children’s ability to care for themselves. Another possibility is that young people may struggle or feel hesitant to communicate certain needs to their parents and will only do so if specifically asked (i.e. when completing a structured needs assessment for example). Self-reports by young people may reflect behaviours that occur across a variety of settings and situations (home, school, with peers) whilst reports by parents are more likely to be based on observations in more circumscribed conditions (either home or school). Adolescents and young adults may also be more reliable informants concerning their mental health problems and the risk they pose to others than their parents (Angold et al. 1987; Cadman et al. 2012). While not all reports of needs in areas such as mental health or risk to others are valid or should result in automatic interventions, these should nevertheless be recorded and followed-up to examine the extent and validity of such needs in more detail. There are relatively high levels of suicide among young males and contact with the criminal justice system reported elsewhere (Lichtenstein et al. 2012; Young et al. 2003) which stresses the importance of identifying and examining self- and parent reported needs which may have potentially far-reaching and long-term negative consequences (O’Brien et al. 2016).

The present study found moderate or substantial agreement in only 4 out of the 25 domains considered. These included *eyesight/hearing, mobility, seizures* and *major mental health problems* indicating that young people and parents agreed predominantly in areas of physical health needs but not in others (although young people with ADHD rated higher needs in *eyesight/hearing* than parents). Thus, the level of agreement found in the present study was considerably lower than previously reported (Hansson et al. 2001; Slade et al. 1998).

### Implications for Service Development

Given the current drive towards needs-led service delivery for young people and adults with mental health problems and the growing evidence suggesting that needs-led services are associated with better outcomes, instruments that reliably identify patient needs are needed in routine clinical practice. We found poor agreement and different types of need being reported by the young person and parent suggesting that both perspectives need to be considered. An awareness of differences in young person and parent perspectives of need may facilitate more effective service planning by ensuring that a more comprehensive picture of the young person’s needs is achieved. These two perspectives could be monitored over time to review what is, and what is not, effective with this group, so that resources are provided for effective interventions.

### Patient Involvement

The finding that adolescents and young adults with neurodevelopmental disorders identify fewer needs than their parents highlights the difficulties presented by this difference in view. The UK Department of Health (2010a, b) has advocated for patients to be encouraged to participate in the assessment of their needs and for young people’s needs to be listened to and placed at the heart of care planning (Department of Health 2010a). An attempt should be therefore be made by service providers to include the views of young people overcoming the difficulties arising from the combination of cognitive impairment, physical problems and/or mental state abnormalities.

Assessing needs directly from young people may be particularly crucial when investigating why people with ADHD and ASD use (or do not) services given that their own assessment of their needs is likely to be a strong determinant of whether or not they seek help from services. Current evidence suggests that young people with neurodevelopmental disorders are not utilizing services to their fullest extent and are not generally given the support that will foster successful transition from child to adult mental health services (Eklund et al. 2016; Nathenson and Zablotsky 2017; Singh et al. 2010). Future research should seek to determine what factors are related to service utilization among this group and to what extent young people with ASD and ADHD are reporting needs that are not being met. Given the reported increased risk of mortality among individuals with ASD (Hirvikoski et al. 2016), more effort should also be made to effectively engage and involve young people with ASD in service design and delivery.

### Carer Perspective

The contribution of informal carers in mental health and learning disability care is now being increasingly acknowledged. In the majority of domains, caregivers reported significantly higher amounts of need than their children. In some particular domains, the discrepancy was very large (e.g. food, looking after the home, exploitation risk, money management).

Given the known issues with introspection and self-reflection in autism and ADHD, the finding that parents reported more needs than young people stresses the importance of seeking parental opinion of needs. Parents may correctly perceive a problem in areas such as self-care and home-care, where their children do not, due to an increased awareness of how their child’s behaviour compares to those of typically developing youth of the same age. The view that their adolescent or young adult child may have problems with self-care and home-care may be a significant concern for parents when placed in the context of their growing child’s ability to care for themselves or live independently in the future.

Parents of many individuals with ASD or ADHD continue to be an important source of support and therefore addressing the parents’ needs is also important. Previous studies have identified that parents among this group often present with high levels of stress and caregiver burden and therefore meeting their child’s needs in areas such as self-care and home-care may add to this stress. Therefore, finding ways to provide support, education, resources, and other interventions in areas of need where parents worry will go a long way to reducing risk of a bad outcome, and relieving parent stress, helping the individual participate in society, and achieve a much better quality of life.

It is important that clinicians recognise that young people and parents may disagree on needs and work with families to build a shared understanding about young people’s needs. By doing so both the young person’s and parent’s perspectives are acknowledged and information is shared making it more likely that clinical interventions are supported (Lasalvia et al. 2012).

### Study Limitations

There may be some underestimation of need as the ADHD sample was originally recruited from clinics and comprised those with a childhood IQ above 70. Prior contact with child and adolescent mental health services among the ADHD sample may have resulted in a higher likelihood of service contact at follow-up which, in turn, may have led to a higher number of met needs in this sample. Given that ADHD symptoms and impairments have been reported to be higher among those with ADHD with intellectual disabilities (ID) than among those with ADHD without ID (Xenitidis et al. 2010), it is likely that this further contributed to the lower levels of needs reported among the ADHD sample.

In addition, as this study consisted mainly of males it is not possible to say how representative the current findings are for females with neurodevelopmental disorders. We recommend that future studies seek to establish what the needs are of females among this group and how female adolescents’ and young adults’ perceptions of needs may differ from perceptions of needs among their parents.

## Conclusion

Service providers are charged with the task of enabling adolescents and young adults with neurodevelopmental disorders to access services that are designed around their individual needs, with fast and convenient care delivered to a consistently high standard, and with additional support where necessary (Jivanjee et al. 2008; Department of Health 2011). To achieve this aim it must look after the individual needs of these groups and their carers. Both parent and young people’s perspectives of need should be assessed to reduce the likelihood of needs going unidentified and unaddressed.
